# Effects of Varying Dietary DL-Methionine Levels on Productive and Reproductive Performance, Egg Quality, and Blood Biochemical Parameters of Quail Breeders

**DOI:** 10.3390/ani10101839

**Published:** 2020-10-09

**Authors:** Fayiz M. Reda, Ayman A. Swelum, Elsayed O.S. Hussein, Shaaban S. Elnesr, Ahmad R. Alhimaidi, Mahmoud Alagawany

**Affiliations:** 1 Poultry Department, Faculty of Agriculture, Zagazig University, Zagazig 44519, Egypt; fayizreda@yahoo.com; 2 Department of Animal Production, College of Food and Agriculture Sciences, King Saud University, Riyadh 11451, Saudi Arabia; shessin@ksu.edu.sa; 3 Department of Theriogenology, Faculty of Veterinary Medicine, Zagazig University, Sharkia 44519, Egypt; 4 Department of Poultry Production, Faculty of Agriculture, Fayoum University, Fayoum 63514, Egypt; ssn00@fayoum.edu.eg; 5 Department of Zoology, College of Science, King Saud University, Riyadh 11451, Saudi Arabia; AhmedAl-Himadi@ksu.edu.sa

**Keywords:** DL-methionine, production, reproduction, antioxidant, immunity, egg quality, quails

## Abstract

**Simple Summary:**

This study investigated the effects of different DL-methionine levels on quail breeders kept from 8–16 weeks of age. The results revealed that using DL-methionine at levels of 0.5 or 1.5 g/kg improved the liver and kidney functions, lipid profile, immunity and antioxidant parameters of quail breeders.

**Abstract:**

The present study was carried out to study the effects of varying dietary DL-methionine (0, 0.5, 1.5, 2.5, and 3.5 g/kg) levels on the productive and reproductive performance, egg quality and blood biochemical parameters of quail breeders. In total, 150 mature Japanese quails at eight weeks of age were randomly allotted to five groups of 30 for each group. Each group included five replicates, each of six quails (four females and two males). The results showed that egg number, egg weight and egg mass were higher (*p <* 0.05) with the addition of all DL-methionine levels than that of the control group. Quails from the control group had a lower feed intake (*p* < 0.001) and a worse feed conversion ratio (FCR) than those from the DL-methionine-treated groups. Supplementation of DL-methionine up to 2.5 g/kg in quail diets increased fertility and hatchability percentages. Birds fed DL-methionine at 1.5 g/kg had the best egg production indices, better FCR and the highest values of fertility and hatchability. Egg weight, yolk %, Haugh unit, egg shape index and unit surface shell weight (USSW) were increased and eggshell % was decreased in quail supplemented with DL-methionine levels compared with the control quail (*p <* 0.05). Dietary DL-methionine levels did not affect (*p* > 0.05) the hemoglobin (Hb), red blood cells (RBCs), white blood cells (WBCs) and packed cell volume (PCV) of quails. DL-methionine levels (0.5 and 2.5 g/kg) augmented lymphocytes and basophile (*p <* 0.05). Low DL-methionine levels (0.5 or 1.5 g/kg) improved liver enzymes and kidney functions. Dietary DL-methionine levels (except 3.5 g/kg) declined serum lactate dehydrogenase (LDH) and decreased lipid profile parameters (except high-density lipoprotein—HDL). Supplementation of DL-methionine at 0.5 and 1.5 g/kg increased immunoglobulin (IgG, IgM and IgA) and superoxide dismutase (SOD), total antioxidant capacity (TAC), catalase (CAT) and reduced glutathione (GSH) (*p <* 0.001) compared with the control. In conclusion, dietary supplementation of DL-methionine (1.5 g/kg) can enhance the reproductive performance and egg quality of quail breeders. DL-methionine use at levels of 0.5 or 1.5 g/kg improved the liver and kidney functions, lipid profile, immunity and antioxidant parameters of Japanese quail.

## 1. Introduction

The best strategy to optimize production and reproduction in poultry species while also mitigating the harmful results of environmental conditions is proper nutrition [1,2,3]. One of the pillars of nutrition is the use of amino acids in poultry diets. Methionine represents the first limiting amino acid in broilers. Bunchasak [4] summarized the several roles of methionine as follows: (1) an essential amino acid for the synthesis of protein; (2) a sulfur donor; (3) an amino acid involved in the synthesis of polyamine; (4) a precursor of main intermediates in metabolic pathways, for instance, carnitine or cystine; and (5) a methyl donor group for the normal formation of co-enzyme S-adenosylmethionine and normal cellular metabolism. Elnesr et al. [5] stated that methionine’s main function is as an antioxidant, and reported that the improvement in the antioxidant system activity is one of the solutions available to increase productivity in the poultry industry. Synthetic sources of methionine such as DL-methionine (DL-Met) are included in poultry feed to optimize the dietary level of methionine. Methionine plays an essential role in energy production and boosts the livability, performance and feed efficiency utilization in poultry [2,6]. Kidd et al. [7] pointed out that healthy poultry responded positively to the inclusion of amino acids as feed additives and had an affirmative impact on performance. Methionine supplementation can alter the immune response and is beneficial in reducing immunologic stress [8]. The addition of methionine boosted the reproduction performance, egg quality and egg production of broiler breeders [9,10].

An adequate amount of methionine must be introduced to meet the nutritional and physiological requirements to maintain the health and performance of birds [11,12]. Thus, updating the nutritional supplies of modern Japanese quails may be an imperative approach in commercial rearing systems. Abou-Kassem [13] found that the performance was significantly improved for quails fed a diet containing a methionine level higher than the recommended level. It is hypothesized that the use of high methionine levels in diets is expected to have beneficial impacts on quail breeders. Therefore, this study aimed to study the effects of different dietary DL-methionine levels on the productive and reproductive performance, egg quality, hematology, liver and kidney function, lipid profile, and antioxidant and immune parameters of quail breeders (8–16 weeks of age).

## 2. Materials and Methods

### 2.1. Experimental Design and Animal Husbandry

In total, 150 mature Japanese quails at eight weeks of age (100 females and 50 males) were used. The present study was carried out at the Poultry Department, Faculty of Agriculture, Zagazig University, Zagazig, Egypt, to study the effects of varying dietary DL-methionine levels (0, 0.5, 1.5, 2.5, and 3.5 g/kg) on the productive and reproductive performance, egg quality and blood biochemical parameters of quail breeders. Birds were randomly allotted to five treatment groups of 30 for each group. Each group included five replicates, each of 6 quails (4 females and 2 males). The basal ration (Table 1) was formulated to meet quail requirements. Quails were reared in conventional-type cages (50 × 30 × 50 cm^3^ of floor space) with water and feed provided ad libitum. Quails were exposed to a 17-h light/7-h dark cycle during the experiment. The experimental procedures were performed according to the Local Experimental Animal Care Committee. The ethical approval code is ZU-IACUC/2/F/95/2018.

### 2.2. Data Collection

Data collection began 4 weeks after feeding the quail with the experimental diets (12 weeks old). Feed intake (FI) was recorded weekly, while feed conversion ratio (FCR, g feed/g egg) was computed as the amount of FI divided by the egg mass. Egg weight and egg numbers were recorded every day to compute the egg mass (egg number × egg weight). Monthly egg quality parameters were measured using three eggs per replicate. Egg quality parameters (yolk percentages, albumen, and shell; thick of shell; unit surface shell weight (USSW), shape index of the egg (ESI), and Haugh unit) were measured [14].

### 2.3. Fertility and Hatchability

At 12 and 16 weeks of age, fifty eggs from each treatment were selected and incubated. After hatching, baby quails were counted and non-hatched eggs were checked and broken to compute the % of fertility and hatchability [15].

### 2.4. Blood Parameters

After slaughter, blood samples were randomly collected from 5 birds per treatment into heparinized tubes. Hematological parameters were measured. Hematological parameters (white blood cells (WBCs), mid-range (MID), granulocytes (GRA), lymphocytes (LYM), red blood cells (RBCs), hemoglobin (HGB), mean corpuscular hemoglobin (MCH), mean corpuscular volume (MCV), hematocrit (HCT), and platelet count (PLT)) were determined. Regarding biochemical parameters, blood samples were centrifuged (G force rate = 2146.56× *g*) for 900 s. Plasma total protein (g/dL), globulin (g/dL), albumin (g/dL), aspartate transaminase (AST; IU/L), alanine transaminase (ALT; IU/L), lactate dehydrogenase (LDH, IU/L), creatinine (mg/dL), urea (mg/dL), total cholesterol (TC; mg/dL), triglyceride (TG; mg/dL), high-density lipoprotein (HDL) cholesterol (mg/dL), and low-density lipoprotein (LDL) cholesterol (mg/dL) levels were determined spectrophotometrically using commercial kits from Biodiagnostic Company (Giza, Egypt). The level of superoxide dismutase (SOD), reduced glutathione (GSH), catalase (CAT), total antioxidant capacity (TAC), and malondialdehyde (MDA) were determined in plasma using commercial kits and a spectrophotometer (Shimadzu, Japan). Levels of immunoglobulin G (IgG; mg/dL), A (IgA; mg/dL) and M (IgM; mg/dL) and lysozyme (mg/dL) were also measured using commercial kits.

### 2.5. Statistics

Data on productive and reproductive functions, egg quality criteria, hematology, liver and kidney function, lipid profile, and antioxidant and immune parameters of quail breeders were analyzed by a generalized linear model using a normal distribution and the identity link function with the Statistical Analysis System (SAS; SAS Institute, Cary, NC, USA) [16].

## 3. Results

### 3.1. Productive Performance

Egg production results are presented in Table 2. Egg number, egg weight, egg mass were significantly (*p <* 0.05) increased (linear and quadratic) by dietary inclusion of all DL-methionine levels throughout the experimental periods (8–12, 12–16 and 8–16 weeks). Birds fed DL-methionine at 1.5 g/kg had the best egg production indices.

As noted in Table 2, quails from the control group had a lower feed intake (*p <* 0.001, linear) than those from the DL-methionine-treated groups at 8–12 and 8–16 weeks of age. Birds fed DL-methionine at 3.5 g/kg consumed more feed at 8–12 weeks of age. Throughout the whole period (8–16 weeks), the groups fed DL-methionine at 1.5, 2.5 and 3.5 g/kg recorded a higher feed intake (linear, *p <* 0.001) than the control group and birds fed DL-methionine at 0.5 g/kg. However, at 12–16 weeks of age, the group fed DL-methionine at 1.5 g/kg consumed more feed than the control and other treated groups. Throughout all periods, the control group had a worse FCR than all treated groups (linear and quadratic, *p <* 0.05). Among treated groups, the group fed DL-methionine at 1.5 g/kg had a better FCR in all periods.

### 3.2. Fertility and Hatchability Percentage

The effects of dietary DL-methionine supplementation on reproductive attributes, including fertility and hatchability, are presented in Table 3. The supplementation of the diet with all levels of DL-methionine increased (quadratic, *p <* 0.05) fertility and hatchability throughout the experimental periods (8–12, 12–16 and 8–16 weeks). The group fed DL-methionine at 1.5 g/kg recorded the highest values of fertility and hatchability.

### 3.3. Egg Quality

As indicated in Table 4, egg weight, yolk %, Haugh unit and USSW increased linearly and quadratically (*p <* 0.05) in laying quail supplemented with DL-methionine at any level compared with the control quail. Albumin % was increased quadratically (*p <* 0.05) with the addition of DL-methionine at 0.5 or 1.5 g/kg in the quail diet compared with the control and other treated groups. However, the inclusion of all levels of DL-methionine (except 0.5 g/kg) in the quail diet decreased the eggshell % (quadratic, *p <* 0.05) and shell thickness (linear, *p <* 0.001) compared with the control group. Egg shape index was increased (linear, *p <* 0.05) in the groups fed DL-methionine at levels of 0.5, 1.5 and 3.5 g/kg compared with the group fed DL-methionine (2.5 g/kg) and the control group. Dietary DL-methionine level did not affect the yolk index.

### 3.4. Hematology

The effects of the addition of DL-methionine on the blood hematology of laying quail are presented in Table 5. Dietary DL-methionine levels did not affect (*p* > 0.05) the Hb, RBCs, WBCs, PCV %, MCV, MCH, MCHC, eosinophil %, segmented forms % or monocyte % of laying quail. Platelet count was increased (linear, *p <* 0.05) by the addition of DL-methionine at 0.5, 2.5 and 3.5 g/kg; basophile % was augmented (linear, *p <* 0.05) by the addition of DL-methionine at any level compared with the control group. The supplementation of the diet with of DL-methionine at levels of 0.5 and 3.5 g/kg quadratically increased (*p <* 0.05) band forms (staff) %, while dietary levels of 0.5 and 2.5 g/kg quadratically augmented (*p <* 0.05) lymphocyte % compared with the control group.

### 3.5. Liver and Kidney Function

The liver and kidney functions were shown in Table 6. Serum total protein, albumin and albumin/globulin (A/G) ratio in the treatment groups did not undergo any change accompanying the increasing DL-methionine levels, but the group fed DL-methionine (except 3.5 g/kg) had higher serum globulin than the control group. The highest level of serum AST of the quail was detected in the group treated with DL-methionine (2.5 and 3.5 g/kg) in comparison with the control and other treated groups (linear, *p <* 0.001). The highest level of serum ALT was detected in the 3.5 g/kg DL-methionine-treated group compared with the control and other treated groups (linear and quadratic, *p <* 0.05). Dietary DL-methionine levels (except 3.5 g/kg) declined serum LDH quadratically (*p <* 0.001) compared with the control group. The lowest creatinine levels were observed in groups fed DL-methionine at 0.5 and 1.5 g/kg, and the lowest urea levels were observed in groups fed DL-methionine at 0.5 g/kg, but the greatest values of creatinine and urea were detected in the group fed DL-methionine at 3.5 g/kg (quadratic, *p <* 0.05).

### 3.6. Lipid Profile

Lipid profile data are presented in Table 7. Total cholesterol and LDL levels decreased with dietary DL-methionine levels. Triglycerides and very low-density lipoprotein (VLDL) levels declined with dietary DL-methionine levels (except 3.5 g/kg) compared with the control group. However, HDL levels increased (linear and quadratic, *p <* 0.05) due to the inclusion of all concentrations of DL-methionine in the laying quail diet.

### 3.7. Antioxidant and Immunity Indices

As shown in Table 7, the levels of IgG, IgM and IgA were significantly increased (linear and quadratic, *p <* 0.001) in DL-methionine-treated groups compared with those in the control. The group fed DL-methionine at 0.5 g/kg recorded the highest values of lysozyme. The activities of SOD, TAC, CAT and GSH were increased quadratically (*p <* 0.05) by the dietary supplementation of DL-methionine at 0.5 and 1.5 g/kg compared with those in the control. The dietary DL-methionine levels (except 2.5 g/kg) quadratically decreased (*p <* 0.001) MDA levels.

## 4. Discussion

Methionine supplementation of the basal diet improved the egg production of quail breeders [17]. Similarly, Liu et al. [18] and Bunchasak and Silapasorn [19] stated that the addition of DL-Met in the basal diet improved broiler breeders’ egg weight and hen-day egg production. Kalvandi et al. [20] illustrated that supplementation of methionine increased egg weight and egg production compared to the basal group. Meng et al. [21] clarified that breeders’ egg weight and laying rate were significantly augmented after the addition of methionine in the diet. The explanation could be that methionine supplementation augments protein deposition, thus encouraging egg production [22]. Gonzalez-Esquerra and Leeson [23] exhibited that supplementation of methionine in the diet boosts the performance through polyamine metabolism pathways.

Kalvandi et al. [20] illustrated that supplementation of methionine increased the daily feed intake and improved the feed conversion ratio compared to the basal group. Meng et al. [21] elucidated that breeders’ feed conversion ratios were significantly improved after the addition of methionine in diet. The improvement of feed efficiency with methionine levels may be elucidated by the fact that the nutrient requirement of quail based on National Research Council (NRC) [22] are different from the present commercial birds. These birds have a better performance than quail because of the management practice and genetic selection [23]. Thus, birds require more amino acids to meet their requirements.

The supplementation of DL-methionine significantly enhanced the hatching rate and fertility rate compared to the control group [10]. Quails fed with methionine diets showed higher hatchability and fertility than those fed the basal diet [20]. The current results showed that Met has an affirmative role in encouraging reproduction performance. This may be due to the role of Met in the synthesis of a glutathione precursor that can clear reactive oxygen species (ROS) and then alleviate the harmful impacts of ROS on the protein, lipid, and DNA structures. The excessive ROS created in the embryo tissues cause deleterious influences, leading to high embryo mortality. Thus, the supplementation of Met can boost the antioxidant indices of the chick embryo and increase the hatchability [10,20].

Moreover, the methionine requirement determined by NRC [22] is for quail breeders and this may be insufficient for supporting their productive and reproductive performance (especially under hyper-thermo neutral conditions). It has been shown that the dietary addition of methionine above NRC [22] recommendations improved cellular immunity in birds [10,12].

It is well known that methionine has favorable effects on the physiology, metabolism of egg production, egg quality and general health status of birds. Eggshell quality is a major concern for egg producers and consumers alike [9,11,12]. The current study stated that Met improved the egg quality of laying quail, in agreement with Xiao et al. [10], who indicated that the supplementation of methionine enhanced eggshell strength, relative eggshell weight and thickness compared with the control group, but did not affect albumen height, egg shape index and Haugh unit. Kalvandi et al. [20] clarified that the supplementation of methionine in quail diets increased eggshell thickness and Haugh unit (HU) score, but did not affect the yolk and albumen percentages. The improvement of egg quality in groups fed different levels of Met may be due to the fact that Met enhance the antioxidant performance within the body [24,25].

Dietary DL-methionine levels did not affect most hematological indices. These results are in agreement with studies by Zhang [26], who stated that Met supplementation did not influence packed cell volume, red blood cell or white blood cell differentiation counts. However, Kalvandi et al. [20] clarified that breeder quails that received Met treatments had higher lymphocyte and lower heterophil and heterophil/lymphocyte (H/L) ratios than quails fed a basal diet, but eosinophil and monocyte did not differ among treatments. The levels of methionine treatments led to a significant decrease in heterophils and an increase in blood lymphocytes and heterophil/lymphocyte ratio as a stress index [27].

Biochemical indicators in the blood can be used to display the health status of poultry. The concentrations of total protein, globulin, albumin, ALT and AST can be indicators of hepatic function, while uric acid can provide signs about renal function. Blood protein in the current study was similar to the results of Hadinia et al. [28], who revealed that dietary Met levels increased levels of globulin, producing no alterations in total protein concentrations. Xiao et al. [10] stated that Met declined uric acid concentration in serum. This may be attributed to the antioxidant and anti-hepatotoxic activities of DL-methionine. 

Most investigations of avian species indicated that Met supplementation affected apolipoproteins and lipids in the blood [29,30]. Methionine is associated with lipid metabolism, including fatty acid oxidation, steatosis and de novo lipogenesis [31,32]. Hydrophobic lipids (free fatty acids, cholesteryl esters and triacylglycerols) during the laying cycle are produced and collected to form egg yolk precursors, including vitellogenin particles and very low-density lipoprotein [30]. Thus, it would be better to supply Met in laying hens’ diets. The addition of Met in quail diets increased plasma levels of HDL–cholesterol and declined triglycerides total cholesterol and LDL–cholesterol [20]. Met supplements facilitate efficient lipid metabolism in the liver and its transportation to the tissues of birds [33]. The potential mechanisms of the lipid-depressing impacts of Met might be correlated to its antioxidant properties.

Previous studies have displayed that birds’ immune systems are stimulated by dietary Met levels that far exceed the levels recommended to meet birds’ performance needs [24,34]. Supplemental methionine can stimulate immune responses [35]. The immune response was improved with the supplementation of Met by enhancing the proliferation of immune cells and antibodies [36]. Bouyeh et al. [27] indicated that methionine plays four main roles related directly or indirectly to immune system responses, as follows: (1) methionine participates in the synthesis of protein; (2) methionine is a glutathione precursor, a tripeptide, which decreases ROS and thus protects cells from oxidative stress; (3) methionine is required for the polyamine (spermine and spermidine) synthesis that take part in the nucleus and cell division events; and (4) methionine is the most important methyl group donor for the methylation reactions of DNA and other molecules.

Methionine plays a part in the main functions in the body, including ROS elimination and GSH precursors [37], and thus methionine is sensitive to oxidative modification [38,39,40]. The results of previous investigations pointed out that augmented dietary Met levels exerted antioxidant impacts in poultry. The current results confirmed these studies. Swennen et al. [41] denoted that Met enhanced the activities of essential antioxidant enzymes (glutathione peroxidase (GSH-Px) and SOD) in blood. Zhang et al. [42] indicated that increasing methionine levels above NRC recommendations led to an increase in total glutathione in the blood. Moreover, Park et al. [43] stated that the supplementation of different methionine levels increased the total glutathione level and reduced MDA in plasma. Lai et al. [44] showed that the addition of dietary methionine levels augmented GSH-Px activity in chicken serum. Furthermore, supplementation of DL-2-hydroxy-4-methylthiobutanoic acid (HMTBA) as a source of methionine improved the activities of glutathione peroxidase, the total antioxidant capacity, and the concentration of reduced glutathione in duck muscles compared with DLM [45]. On the other hand, both DL-HMTBA and DLM increased the concentrations of MDA in the muscles compared with a basal diet [45].

## 5. Conclusions

Dietary supplementation of DL-methionine (1.5 g/kg) can enhance egg production traits, reproductive performance and egg quality, also improved liver and kidney function, lipid profile, immunity, and antioxidant parameters of Japanese quail. Therefore, it could be concluded that 1.5 g/kg of DL-methionine was the optimal concentration found under our experimental conditions and this level could change depending on the circumstances.

## Figures and Tables

**Table 1:** Ingredients and nutrient contents of the basal diet of Japanese quail.

Ingredients	(g/kg)
Yellow corn	602
Soybean meal (44%)	250
Oil	15
Corn gluten meal, 60%	57
Dicalcium phosphate	13
Limestone	55
Nacl	3
L-lysine	2
DL-methionine	0
Premix *	3
Composition (g/kg)	
Protein	199
ME (Kcal/kg)	2918
Ca	25
P available	3.50
Methionine	3.50
Lysine	10.9
Methionine + cysteine	7.10

* Provides per kg of diet: vitamin A, 12,000 I.U; vitamin D3, 5000 I.U; vitamin E, 130.0 mg; vitamin K3, 3.605 mg; vitamin B1 (thiamin), 3.0 mg; vitamin B2 (riboflavin), 8.0 mg; vitamin B6, 4.950 mg; vitamin B12, 17.0 mg; niacin, 60.0 mg; D-biotin, 200.0 mg; calcium D-pantothenate, 18.333 mg; folic acid, 2.083 mg; manganese, 100.0 mg; iron, 80.0 mg; zinc, 80.0 mg; copper, 8.0 mg; iodine, 2.0 mg; cobalt, 500.0 mg; and selenium, 150.0 mg.

**Table 2:** Productive performance of Japanese quail as affected by dietary DL-methionine.

Items	DL-Methionine Levels					SEM	*p* Value	
	0	0.5	1.5	2.5	3.5		Linear	Quadratic
Egg number/bird								
8–12 weeks	20.67 ^d^	26.33 ^ab^	27.67 ^a^	25.33 ^bc^	24.67 ^c^	0.467	<0.001	<0.001
12–16 weeks	21.33 ^d^	26.17 ^b^	28.00 ^a^	26.67 ^ab^	23.33 ^c^	0.470	0.02	<0.001
8–16 weeks	21.00 ^d^	26.25 ^b^	27.83 ^a^	26.00 ^b^	24.00 ^c^	0.344	<0.001	<0.001
Egg weight (g)								
8–12 weeks	11.45 ^c^	11.83 ^b^	12.57 ^a^	12.44 ^a^	12.43 ^a^	0.068	<0.001	<0.001
12–16 weeks	11.83 ^c^	12.20 ^b^	12.63 ^a^	12.57 ^a^	12.50 ^a^	0.054	<0.001	<0.001
8–16 weeks	11.64 ^c^	12.02 ^b^	12.60 ^a^	12.51 ^a^	12.46 ^a^	0.054	<0.001	<0.001
Egg mass g/bird								
8–12 weeks	236.56 ^c^	311.50 ^b^	347.84 ^a^	315.19 ^b^	306.70 ^b^	5.464	<0.001	<0.001
12–16 weeks	252.31 ^d^	319.30 ^b^	353.83 ^a^	335.23 ^ab^	291.57 ^c^	5.955	<0.001	<0.001
8–16 weeks	244.43 ^d^	315.40 ^b^	350.84 ^a^	325.21 ^b^	299.14 ^c^	4.581	<0.001	<0.001
Feed intake (g/bird)								
8–12 weeks	823.00 ^e^	847.00 ^d^	868.00 ^c^	900.33 ^b^	919.33 ^a^	4.81	<0.001	0.95
12–16 weeks	827.67 ^b^	863.00 ^ab^	893.33 ^a^	851.67 ^b^	838.33 ^b^	11.08	0.79	<0.001
8–16 weeks	825.67 ^c^	855.33 ^b^	881.00 ^a^	876.33 ^a^	879.00 ^a^	5.63	<0.001	<0.001
Feed conversion ratio (g feed/g egg)								
8–12 weeks	3.48 ^a^	2.72 ^c^	2.49 ^d^	2.86 ^c^	3.00 ^b^	0.03	<0.001	<0.001
12–16 weeks	3.28 ^a^	2.70 ^c^	2.53 ^d^	2.54 ^d^	2.87 ^b^	0.02	<0.001	<0.001
8–16 weeks	3.38 ^a^	2.71 ^c^	2.51 ^d^	2.70 ^c^	2.94 ^b^	0.02	<0.001	<0.001

^a, b, c, d, e^ Means in the same row with no superscript letters after them or with a common superscript letter following them are not significantly different (*p* < 0.05). SEM: standard error of the mean.

**Table 3:** Fertility and hatchability % of Japanese quail as affected by dietary DL-methionine.

Items	DL-Methionine Levels					SEM	*p* Value	
	0	0.5	1.5	2.5	3.5		Linear	Quadratic
Fertility %								
8–12 weeks	72.78 ^c^	89.18 ^ab^	94.66 ^a^	87.53 ^ab^	80.98 ^bc^	2.533	0.11	<0.001
12–16 weeks	75.49 ^d^	89.44 ^ab^	93.47 ^a^	84.72 ^bc^	78.05 ^cd^	1.934	0.96	<0.001
8–16 weeks	74.13 ^d^	89.31 ^ab^	94.07 ^a^	86.12 ^bc^	79.52 ^cd^	2.074	0.32	<0.001
Hatchability %								
8–12 weeks	64.45 ^d^	80.74 ^b^	85.98 ^a^	72.58 ^c^	67.52 ^cd^	1.530	0.70	<0.001
12–16 weeks	67.28 ^d^	83.06 ^ab^	89.03 ^a^	76.11 ^bc^	71.67 ^cd^	2.233	0.80	<0.001
8–16 weeks	65.86 ^d^	81.89 ^b^	87.51 ^a^	74.34 ^c^	69.60 ^cd^	1.564	0.99	<0.001

^a, b, c, d^ Means in the same row with no superscript letters after them or with a common superscript letter following them are not significantly different (*p* < 0.05). SEM: standard error of the mean.

**Table 4:** Egg quality of Japanese quail as affected by dietary DL-methionine.

Items	DL-Methionine Levels					SEM	*p* Value	
	0	0.5	1.5	2.5	3.5		Linear	Quadratic
Egg Weight (g)	12.28 ^c^	13.97 ^ab^	14.49 ^a^	13.49 ^b^	13.55 ^b^	0.187	0.01	<0.001
Yolk %	30.61 ^c^	31.62 ^b^	32.20 ^ab^	32.58 ^a^	32.78 ^a^	0.177	<0.001	0.03
Albumin %	54.42 ^b^	56.35 ^a^	56.21 ^a^	54.85 ^b^	54.94 ^b^	0.267	0.65	<0.001
Eggshell %	14.97 ^a^	12.03 ^b^	11.59 ^b^	12.57 ^b^	12.28 ^b^	0.359	0.002	<0.001
Shell thickness (mm)	0.164 ^d^	0.170 ^d^	0.177 ^c^	0.183 ^b^	0.195 ^a^	0.002	<0.001	0.18
Egg shape index	70.67 ^b^	80.02 ^a^	80.96 ^a^	75.12 ^ab^	80.75 ^a^	2.088	0.05	0.11
Yolk index	43.93	45.75	48.30	46.13	46.49	0.884	0.09	0.05
Haugh unit	81.20 ^c^	83.32 ^b^	84.08 ^b^	85.19 ^a^	85.17 ^a^	0.301	<0.001	0.01
USSW(g/cm^2^)	46.99 ^c^	48.52 ^ab^	48.97 ^a^	48.10 ^b^	48.16 ^b^	0.166	0.01	<0.001

USSW = unit surface shell weight (mg/cm^2^). ^a, b, c, d^ Means in the same row with no superscript letters after them or with a common superscript letter following them are not significantly different (*p* < 0.05). SEM: standard error of the mean.

**Table 5:** Hematology of Japanese quail as affected by dietary DL-methionine.

Items	DL-Methionine Levels					SEM	*p* Value	
	0	0.5	1.5	2.5	3.5		Linear	Quadratic
Hemoglobin	9.20	11.00	11.50	10.07	10.07	0.849	0.77	0.11
RBCs	2.45	2.76	2.99	3.16	2.67	0.344	0.53	0.30
WBCs	16.00	19.06	20.8	18.21	17.77	1.346	0.56	0.06
Platelet count	39.67 ^a^	31.50 ^b^	34.33 ^ab^	29.33 ^b^	32.85 ^b^	1.582	0.02	0.052
PCV %	32.39	34.28	34.79	34.11	32.90	1.441	0.88	0.28
MCV	131.65	125.84	125.94	112.04	123.21	8.925	0.41	0.65
MCH	37.59	39.94	41.02	32.47	37.51	2.078	0.41	0.70
MCHC	28.59	31.97	32.98	29.42	30.50	1.594	0.81	0.17
Basophile %	0.16 ^b^	0.24 ^a^	0.21 ^a^	0.24 ^a^	0.23 ^a^	0.017	0.03	0.05
Eosinophil %	0.26	0.42	0.33	0.33	0.42	0.043	0.14	0.82
Band forms (staff) %	0.60 ^c^	0.95 ^a^	0.70 ^bc^	0.69 ^bc^	0.73 ^b^	0.029	0.94	0.01
Segmented forms %	35.70	32.63	34.69	32.90	27.07	2.091	0.15	0.49
Lymphocyte %	62.08 ^c^	64.35 ^ab^	63.15 ^bc^	65.22 ^a^	62.88 ^bc^	0.567	0.23	0.03
Monocyte %	1.20	1.43	0.92	1.29	1.08	0.118	0.35	0.99

^a, b, c^ Means in the same row with no superscript letters after them or with a common superscript letter following them are not significantly different (*p* < 0.05). SEM: standard error of the mean.

**Table 6:** Liver and kidney functions of Japanese quail as affected by dietary DL-methionine.

Items	DL-Methionine Levels					SEM	*p* Value	
	0	0.5	1.5	2.5	3.5		Linear	Quadratic
Total protein (g/dL)	3.34	3.67	3.54	3.59	3.86	0.095	0.01	0.85
Albumin (g/dL)	1.28	1.34	1.41	1.43	1.37	0.040	0.08	0.11
Globulin (g/dL)	2.31 ^d^	3.03 ^ab^	3.23 ^a^	2.76 ^bc^	2.49 ^cd^	0.082	0.78	<0.001
A/G ratio (%)	0.55	0.44	0.44	0.52	0.56	0.030	0.40	0.01
AST (IU/L)	167.60 ^bc^	162.00 ^c^	188.55 ^b^	233.10 ^a^	249.05 ^a^	7.078	<0.001	0.06
ALT (IU/L)	11.08 ^bc^	14.50 ^b^	12.01 ^bc^	10.07 ^c^	22.09 ^a^	0.917	<0.001	<0.001
LDH (IU/L)	334.60 ^b^	267.15 ^c^	224.00 ^d^	218.60 ^d^	378.10 ^a^	9.070	0.24	<0.001
Creatinine (mg/dL)	0.57 ^b^	0.46 ^c^	0.49 ^c^	0.54 ^b^	0.63 ^a^	0.012	0.0008	<0.001
Urea (mg/dL)	1.65 ^b^	0.93 ^c^	1.41 ^bc^	1.59 ^b^	3.10 ^a^	0.131	<0.001	<0.001

^a, b, c, d^ Means in the same row with no superscript letters after them or with a common superscript letter following them are not significantly different (*p* <0.05). albumin/globulin (A/G); Aspartate transaminase (AST); Alanine aminotransferase (ALT); Lactate dehydrogenase (LDH); SEM: standard error of the mean.

**Table 7:** Lipid profile, immunity and antioxidants of Japanese quail as affected by dietary DL-methionine.

Items	DL-Methionine Levels					SEM	*p* Value	
	0	0.5	1.5	2.5	3.5		Linear	Quadratic
Lipid profile								
Total cholesterol (mg/dL)	366.40 ^a^	205.29 ^d^	284.70 ^c^	283.15 ^c^	331.55 ^b^	3.685	0.60	<0.001
Triglycerides (mg/dL)	1094.50 ^b^	407.95 ^e^	810.65 ^d^	990.50 ^c^	1207.00 ^a^	11.004	<0.001	<0.001
HDL (mg/dL)	12.30 ^c^	17.42 ^b^	22.25 ^a^	19.82 ^ab^	16.38 ^b^	1.169	0.03	<0.001
LDL (mg/dL)	135.21 ^a^	106.28 ^b^	100.32 ^b^	65.24 ^c^	73.77 ^c^	4.984	<0.001	0.04
VLDL (mg/dL)	218.90 ^b^	81.59 ^e^	162.13 ^d^	198.10 ^c^	241.40 ^a^	2.201	<0.001	<0.001
Immunity								
IgM (mg/dl)	0.34 ^d^	0.83 ^a^	0.47 ^b^	0.39 ^c^	0.36 ^d^	0.005	<0.001	<0.001
IgG (mg/dl)	0.53 ^d^	1.22 ^a^	0.69 ^b^	0.62 ^c^	0.57 ^d^	0.013	<0.001	<0.001
IgA (mg/dl)	0.33 ^d^	0.69 ^a^	0.42 ^b^	0.38 ^c^	0.34 ^d^	0.005	<0.001	<0.001
Lysozyme (mg/dl)	0.14 ^b^	0.24 ^a^	0.20 ^ab^	0.17 ^b^	0.15 ^b^	0.016	0.47	0.01
Antioxidants								
SOD (U/mL)	0.11 ^c^	0.28 ^a^	0.16 ^b^	0.12 ^c^	0.13 ^c^	0.006	<0.001	<0.001
MDA (nmol/mL)	0.46 ^a^	0.13 ^d^	0.34 ^c^	0.42 ^ab^	0.40 ^b^	0.014	0.01	<0.001
TAC	0.11 ^c^	0.21 ^a^	0.17 ^ab^	0.14 ^cb^	0.12 ^c^	0.011	0.16	<0.001
CAT (ng/mL)	0.13 ^c^	0.22 ^a^	0.18 ^b^	0.15 ^bc^	0.13 ^c^	0.010	0.08	<0.001
GSH (ng/mL)	0.12 ^c^	0.29 ^a^	0.17 ^b^	0.12 ^c^	0.14 ^c^	0.006	<0.001	<0.001

^a, b, c, d, e^ Means in the same row with no superscript letters after them or with a common superscript letter following them are not significantly different (*p* < 0.05). High-density lipoprotein (HDL); Low-density lipoprotein (LDL); Very low-density lipoprotein (VLDL); Immunoglobulin M (IgM); Immunoglobulin G (IgG); Immunoglobulin A (IgA); Superoxide dismutase (SOD), malondialdehyde (MDA); Total antioxidant capacity (TAC); Catalase (CAT); Reduced glutathione (GSH). SEM: standard error of the mean.
